# Gonorrhoea & its co-infection with other ulcerative, non-ulcerative sexually transmitted & HIV infection in a Regional STD Centre

**Published:** 2011-03

**Authors:** Manju Bala, Jhinuk Basu Mullick, Sumathi Muralidhar, Joginder Kumar, V. Ramesh

**Affiliations:** Regional STD Teaching, Training & Research Centre, Vardhman Mahavir Medical College & Safdarjang Hospital, New Delhi 110 029, India

Sir,

Gonorrhoea is one of the most common sexually transmitted infections (STIs) in developing countries and is a global public health problem 1,2.

Gonorrhoea is an easily curable STI, but if remained undetected, untreated infections and co-infections can lead to complications like pelvic inflammatory disease, ectopic pregnancy, tubal factor infertility, adverse pregnancy outcomes in females, and testicular and prostate infections and infertility in males. Also, asymptomatic patients, unaware of their infection, may serve as a reservoir of infection to their partners. Moreover, STIs including non-ulcerative STIs like gonorrhoea potentially increase the risk of both transmission and acquisition of human immunodeficiency virus (HIV) 3.

Socio-economic factors like women’s emancipation, permissiveness, homosexuality, population migration and increased availability of diagnostic facilities have resulted in increasing these STI rates. However, rate of gonorrhoea and other non-ulcerative STIs are difficult to determine because clinical presentation is not specific enough and facilities, materials, or personnel for laboratory based diagnosis are inadequate. Moreover, there is lack of reporting mechanism and reluctance to report STIs to public health authorities.

There are many reports on co-infection between gonorrhoea and chlamydia infection. Little information is available on co-infection of gonorrhoea with other STIs. Keeping this aspect in mind we carried out retrospective analysis of six year data to evaluate the gonococcal infection rate in patients attending male and female STD clinic of the Regional STD Teaching, Training & Research Centre, Safdarjang Hospital, New Delhi, and analysed its association with other ulcerative (syphilis, chancroid, herpes, donovanosis), non-ulcerative STIs [chlamydiasis, trichomoniasis, bacterial vaginosis (BV), candidiasis] and HIV infection. This Regional STD Centre has been monitoring the trends of antimicrobial resistance in *Neisseria gonorrhoeae* and prevalence of STIs^4^–^7^.

A total of 5871 records from male and female patients who visited the Centre between January 2003 to December 2008 were analyzed. Urethritis related symptoms and genital ulcer, and cervical/vaginal discharge and genital ulcer were the main eligibility criteria for males and females, respectively for inclusion in the study. Demographic information, including age, race, gender, and symptoms was also collected.

Standard laboratory procedures were used for the diagnosis of gonorrhoea and other STIs^8^,^9^. Direct urethral/cervical smear and culture on chocolate agar and saponin-lysed blood agar with vancomycin, colistin, nystatin, trimethoprim (VCNT) supplement were carried out to diagnose *N. gonorrhoeae* and the isolates were confirmed by standard methods. For syphilis, dark field examination, VDRL (Venereal Disease Research Laboratory) test (antigen from Serologist to Govt. of India, Kolkata), *Treponema pallidum* haemagglutination assay (Plasmatec TPHA test kit, Hansard Diagnostics, United Kingdom) in VDRL reactive cases and fluorescent treponemal antibody absorption (FTA-ABS) test using FTA-Abs IgG and IgM IFA (Viro-Immun Labor-Diagnostika Gmbh, Oberursel & Virgo, Calbiotec, USA) in sera giving discrepant results in the above two tests were carried out. For herpes progenitalis ulcer smear and IgM HSV-2 ELISA; for donovanosis tissue smears; and for chancroid smear and culture using two medium *i.e.,* GC agar base with iso-vitalex, vancomycin and fetal calf serum and Mueller Hinton agar with iso-vitalex, vancomycin and fetal calf serum from ulcer base were performed. Tests for chlamydial infections included antigen detection by ELISA (Bio-Rad Laboratories, USA) and direct fluorescent antibody (DFA) test (Immuno FA, Orgenics, Israel) and Gram-stained urethral/cervical smear showing four or more polymorphonuclear cells on high-power examination. For diagnosis of trichomoniasis, a direct wet mount examination and culturing on Whittington media; for candidiasis, direct Gram stained smear examination and culture on Saboraud’s dextrose agar, followed by culture confirmation by germ tube test; and for BV, physiological tests (*p*H test >4 and amine tests) and vaginal Gram stained smear (interpretation following Nugent’s criteria), were performed. Besides these tests, presence of HIV 1 and 2 antibodies were determined in the patients by ELISA/Rapid tests, using NACO approved kits, following NACO guidelines 10 after pretest counselling, and written informed consent, followed by post-test counselling.

The differences in percentages were statistically compared by determining standard error of proportions, tested for significance by using Z test. The presence of co-infections was compared by chi-square test.

During the study period, a total of 353 gonorrhoea cases were detected and throughout the study period, gonorrhoea rate was more in males than females. Of the total 353 cases based on culture positivity, 315 males were harbouring gonorrhoea infections, whereas infected females were only 38. The presence of gonorrhoea in males was observed to be higher in 2003-2006 (varying from 9.3 to 12.1%), dropping in 2007 (6.4%). This decrease from 9.3 per cent in 2003 to 6.4 per cent in 2007 was found to be statistically significant (*P*<0.001). However, in 2008 there was a significant substantial rise to 12.4 per cent (*P*<0.001). The occurrence of gonorrhoea was lower in females varying from 0.4 to 3.8 per cent. Patients in 21-30 yr age group accounted for majority of cases of gonorrhoea (56.4%) and 20.7 per cent belonged to 31-40 yr. This was followed by 15-20 yr age group (13.1%) and 41-50 yr (7.4%). Eight cases (2.2%) belonged to 51-70 yr age group.

Among ulcerative STIs, presence of syphilis was highest (36.9%), followed by herpes progenitalis (15.4%). A few cases were reported to have chancroid (0.9%) and donovanosis (0.1%). Of the non-ulcerative STIs, infection by Candida albicans was most frequently observed (23.3%) while BV, *C. trachomatis, Trichomonas vaginalis* accounted for 5.3, 4.2 and 1.8 per cent, respectively. All the ulcerative STIs and genital warts were more common in males. Rate of genital warts and HIV infection was found to be 11.0 and 9.7 per cent.

The Table shows the distribution of various other STIs by aetiological diagnosis among *N. gonorrhoeae* culture positive cases. Overall, 14.4 per cent (51/353) gonorrhoea patients had co-infection with another STIs. Among the male gonorrhoea cases, the most common co-infection was syphilis *i.e.,* 7.0 per cent followed by HIV 2.2 per cent (*P*<0.01). Other minor co-infections were with *C. albicans* 0.9 per cent, *C. trachomatis* 0.9 per cent, chancroid 0.3 per cent and herpes 0.3 per cent (*P*<0.01).

**Table:** Co-infection of gonorrhoea with other sexually transmitted infections (STIs)

	STI	Male No.(%)	Female No.(%)	Total No.(%)

Total *N. gonorrhoeae* culture positive cases			315	38	353
	Syphilis	22 (7.0)	0	22 (6.3)
	Chancroid	1 (0.3)	0	1 (0.3)
Ulcerative STIs	Herpes	1 (0.3)	0	1 (0.3)
	Donovanosis	0	0	0
	*Trichomonas vaginalis*	0	1 (2.6)	1 (0.3)
	*Candida albicans*	3 (0.9)	6 (15.8)	9 (2.5)
Non-ulcerative STIs	Bacterial vaginosis	Not applicable	6 (15.8)	6 (1.7)
	*Chlamydia trachomatis*	3 (0.9)	1 (2.6)	4 (1.1)
	Genital warts	0	0	0
Other STIs	HIV	7 (2.2)	0	7 (2.0)
	Total co-infections	37 (11.7)	14 (36.8)	51 (14.4)

Among the comparatively low prevalent gonorrhoea positive females, no co-infections were found for ulcerative STIs. The highest association in females was with non-ulcerative STIs such as bacterial vaginosis, and *C. albicans i.e.,* 15.8 per cent each (*P*<0.05). Other statistically insignificant co-infections included *C. trachomatis* and *T. vaginalis* 2.6 per cent each. Co-infection with more than two STIs was rare.

The occurrence of gonorrhoea in patients attending this centre has changed since 1990^6^. It remained constant around 13 per cent from 1990 to 1997 and thereafter, there was a significant rise to 19.4 per cent in 2001 and again decreased to 15.4 per cent in 2002^6^. Gonorrhoea rates in the present study dropped from 12.1 per cent in 2004 to 6.4 per cent in 2007. This drop may either be due to actual decreasing rate of gonorrhoea over the years or due to the fact that gradually less number of patients were reporting to the clinic because of easy availability of antimicrobials as a part of syndromic management of STIs in peripheral and private health set ups.

Our study showed maximum cases in the 21-30 yr age group, and among males. The subjects as found from patient’s history were mostly unmarried and hence more prone to visit commercial sex workers. The data support earlier consensus that young adults and adolescents should constitute priority target group in STD control programme.

Gonorrhoea rate was quite low in females in the present study. It may be because most of the female patients were referred from Gynaecology clinic to STD clinic after they did not respond to syndromic management of genital discharges. Besides, most of the female patients were not having purulent/muco purulent cervical discharge and thus were not cases of gonorrhoea. In contrast, in Mumbai study^11^, prevalence was 9.7 per cent in women attending STD clinic. Gonococcal urethritis rate was observed to be 7 per cent in STD clinic in Assam Medical College Hospital, Dibrugarh^12^.

In the present study, in only 4 of 353 (1.1%) cases of gonorrhoea, co-infection with *C. trachomatis* was observed. This finding is in contrast to high association between *N. gonorrhoeae* and *C. trachomatis* (27.2%) observed in a study from Mumbai, India^11^ and as also (range, 9-67%) found in other studies from United States^13^. In a recent study from Italy 14, co-infection with C. trachomatis was detected in 46 per cent patients. Otherwise also, *C. trachomatis* infection prevalence was low (4.5%) in the present study in comparison to above studies resulting in lesser rate of co-infection. This may have been due to use of less sensitive enzyme immunoassay, and DFA test. The newer, more sensitive nucleic acid amplification tests [such as polymerase chain reaction (PCR)] being more expensive could not be used in this study.

Prevalence of BV among gonorrhoea patients was 15.8 per cent in contrast to 3.2 per cent in Indonesia^15^ and 2.2 per cent in USA 16. In both above, population studied was pregenant women. In a community based study from India^17^, association with BV was nil. Association between *N. gonorrhoeae* and *Trichomonas vaginalis* has been observed to be 1, 10 and 11.7 per cent in Indiana, United States and Mumbai respectively^11^,^18^,^19^. This is quite high in comparison to our study (0.3%). High sensitivity of PCR used in these studies in comparison to culture could have attributed to this. This was explained by Seña *et al*^19^ that urine cultures detected *T. vaginalis* infection in 8 per cent, whereas urine PCR demonstrated infection in 70 per cent.

Our study showed the highest rate of co-infection among gonorrhoea positive cases to be with syphilis (6.3%). Similarly, Bozicevic *et al* 20 found it to be 9 per cent in symptomatic heterosexual men. Recently, high rate of co-infection with HIV i.e., 17 per cent were detected in patients infected with N. gonorrhoeae in Kenya^21^ in comparison to our study (2%). In Mumbai study 11, co-infection with HIV was observed to be very high (92.3%), while it was nil in a STD centre in North Eastern State of India^22^.

In this study, only two patients were observed to have multiple co-infections probably because in gonorrhoea the infection is not as prolonged and the symptomatic patients tend to seek treatment at the early stage.

Differences in the method used for diagnosis of STIs and the size and type of population studied may lead to variations in the co-infection rate. High co-infection rate (14.4%) of gonorrhoea with other STIs from 2003-2008 highlight the considerable burden of the disease indicating that appropriate screening measures for STIs must be widely and consistently implemented so as to assure prompt and effective treatment for infected persons and their sexual partners leading to reductions in disease burden.
